# Splenic Torsion in a Patient with Polysplenia Syndrome and Dorsal Pancreatic Agenesis: A Case Report

**DOI:** 10.70352/scrj.cr.25-0380

**Published:** 2025-11-19

**Authors:** Kensuke Kishida, Hiroshi Nemoto, Hideki Sarukawa, Ryu Matsunaga, Hajime Miyaji, Naoki Yazawa, Kunihiko Shimura, Taku Miyamae

**Affiliations:** 1 Ebina General Hospital, Ebina, Kanagawa, Japan; 2 Department of Surgery, Ebina General Hospital, Ebina, Kanagawa, Japan

**Keywords:** polysplenia syndrome, splenic torsion, agenesis of the dorsal pancreas

## Abstract

**INTRODUCTION:**

Partial splenic torsion in polysplenia syndrome is an extremely rare condition. We encountered and performed surgery for a case of partial splenic torsion in a patient with polysplenia syndrome coexisting with agenesis of the dorsal pancreas.

**CASE PRESENTATION:**

A 30-year-old woman with heterotaxy syndrome presented with intermittent epigastric and right lateral abdominal pain persisting for 2 weeks. She was referred to our hospital. Abdominal contrast-enhanced CT revealed multiple spleens on the right side, a right-sided stomach, and agenesis of the dorsal pancreas. One of the 5 spleens showed no contrast enhancement. Suspecting torsion or infarction, we opted for surgical intervention. Intraoperative findings revealed a small spleen with poor coloration and 180-degree torsion, which we removed. Additionally, we identified another poorly anchored spleen and performed splenopexy to secure this wandering spleen.

**CONCLUSIONS:**

Polysplenia syndrome is a rare condition involving multiple spleens in the setting of heterotaxy syndrome—a defect in the left–right axis of the thoracic or abdominal organs without a complete mirror image. Several reports have described polysplenia syndrome coexisting with agenesis of the dorsal pancreas. Including our case, 11 instances of splenic torsion associated with polysplenia syndrome have been reported in English, with the current patient being the oldest. Furthermore, this is the 1st reported case of splenic torsion occurring in the context of polysplenia syndrome with agenesis of the dorsal pancreas. Our patient had 2 notable anatomical characteristics: first, a vascular variation in which the right celiac artery lacked a common hepatic artery and instead formed a common trunk with the superior mesenteric artery and common hepatic artery, resembling Adachi type VI (group 24); and second, a centrally located, unfixed dorsal spleen that underwent torsion. We hypothesize that splenic torsion occurred due to the central spleen’s mobility and its slightly elongated splenic artery.

## Abbreviations

PV
: portal vein
SA
: splenic artery
SMA
: superior mesenteric artery

## INTRODUCTION

Splenic torsion induced by various causes is a rare condition.^1)^ The spleen is normally anchored within the abdominal cavity by several ligaments, including the gastrosplenic, splenorenal, splenocolic, and phrenicosplenic ligaments. Among reported cases of splenic torsion, some involve torsion of a wandering spleen, which results from congenital or acquired abnormalities leading to weakened or deficient suspensory ligaments.^2,3)^ Furthermore, partial splenic torsion in the context of polysplenia syndrome—considered a specific type of wandering spleen—has also been documented, though only 10 such cases have been reported in the English literature to date.^4–13)^ These cases suggest that some spleens in patients with polysplenia syndrome may exist in a partially wandering state. We report a surgical case of partial splenic torsion in a very rare instance of polysplenia syndrome coexisting with agenesis of the dorsal pancreas.

## CASE PRESENTATION

A 30-year-old woman experienced intermittent epigastric and right lateral abdominal pain that had persisted for 2 weeks. Although her family doctor performed CT and esophagogastroduodenoscopy, the cause of her abdominal pain remained unclear. She had been diagnosed with polysplenia syndrome in childhood and had a pacemaker implanted 6 years earlier for bradyarrhythmia in another hospital. She had no history of pregnancy. Because the pain had intensified the day before presentation, she was referred to our hospital. Physical examination revealed significant tenderness in the right lower quadrant without rebound tenderness. Her initial vital signs were nearly normal, and laboratory findings were unremarkable except for elevated C-reactive protein (11.19 mg/dL) and lactic acid (1.20 mmol/L).

**Fig. 1:**
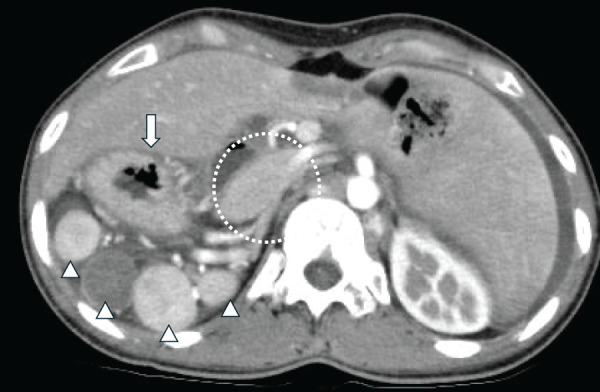
CT findings of the abdomen. This image shows multiple spleens on the right side (white arrowheads), a right-sided stomach (white arrow), a midline liver, and the head of the pancreas without the dorsal pancreas (dotted circle).

**Fig. 2:**
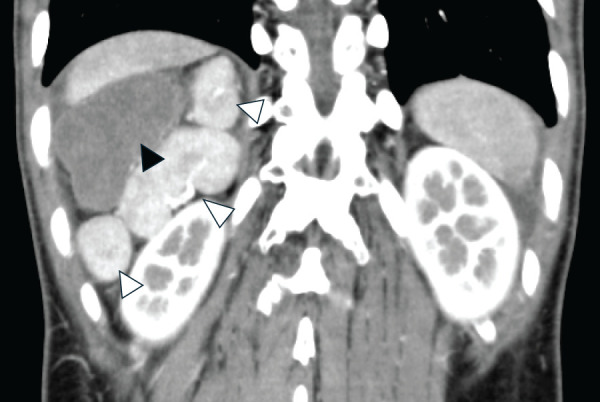
Multiple spleens on CT. In the right upper quadrant of the abdomen, 3 enhanced small spleens are visible (white arrowheads), along with 1 non-enhanced spleen (black arrowhead).

Abdominal contrast-enhanced CT revealed multiple spleens on the right side, a right-sided stomach, midline liver, and agenesis of the dorsal pancreas (**Fig. 1**). One of the 5 spleens showed no contrast enhancement (**Fig. 2**), raising suspicion of torsion or infarction, and we decided to proceed with surgery.

**Fig. 3:**
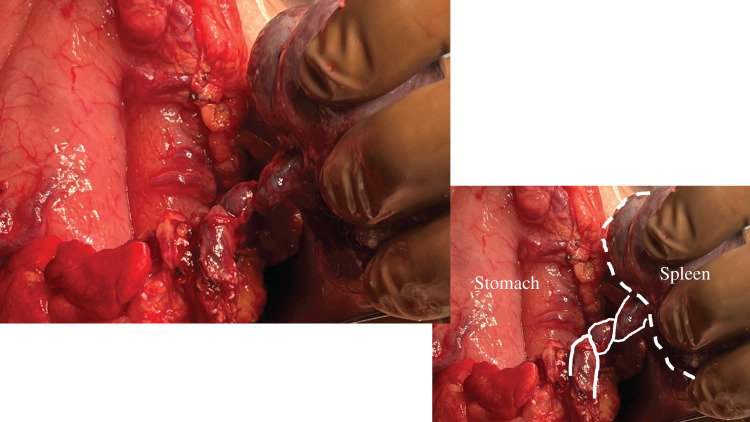
Intraoperative findings revealed splenic torsion.

The laparotomy was performed through an upper midline incision under general anesthesia. The stomach was identified in the upper right abdomen, after which the greater omentum was opened to access the omental bursa. Among the multiple spleens present, 1 small spleen appeared discolored. Because of a 180-degree twist of its splenic vessels, this spleen was ligated and excised at the hilum (**Fig. 3**). Another spleen was found to be poorly anchored. After confirming adequate blood flow with indocyanine green fluorescence imaging, splenopexy was performed using absorbable sutures. The remaining 3 spleens were firmly fixed to the retroperitoneum. The operation lasted 109 minutes, with a total blood loss of 50 mL. The postoperative course was uneventful, and the patient was discharged on the 5th POD.

## DISCUSSION

Heterotaxy syndrome, also known as situs ambiguus, is a defect in the left–right axis arrangement of the thoracic or abdominal organs, without forming a complete mirror image configuration as in situs inversus.^14)^ Right isomerism is characterized by symmetrical development of the right-sided organs and an absence of the spleen, and it is therefore referred to as asplenia syndrome. By contrast, left isomerism involves symmetrical development of the left-sided organs and the presence of multiple spleens and is known as polysplenia syndrome. Although some associated genes have been identified, the exact cause of heterotaxy syndrome remains unknown.^15)^ Numerous cardiovascular abnormalities have been reported in association with left isomerism, including interrupted inferior vena cava, atrioventricular septal defect, and single ventricle, among others.^14)^

Peoples *et al*.^16)^ have reported 146 cases of polysplenia syndrome, in which at least half of the patients had cardiac malformations, and 75% of them died before the age of 5 years. In the absence of severe cardiac abnormalities, however, patients with polysplenia syndrome may survive into adulthood. In polysplenia syndrome, the spleens are typically located along the greater curvature of the stomach and are often situated on the right side.^16,17)^

To date, 11 cases of splenic torsion associated with polysplenia syndrome, including the present case, have been reported in the English-language literature. These patients comprised 9 females and 2 males, ranging in age from the 2nd day of life to 30 years, with only 3 adult cases reported (**Table 1**). The current case represents the oldest patient among them. Detailed information on the patient’s clinical course from birth to the present—particularly concerning the cardiovascular system—was unavailable. However, we believe that the absence of severe congenital heart disease, which likely enabled survival into adolescence, may have contributed to the development of splenic volvulus at this age. Of the 11 patients, 7 had spleens located on the right and 4 on the left. In 7 of the patients for whom the number of spleens was described, 2 had multiple spleens that underwent torsion.^5,11)^ In our case as well, there was a possibility that 2 spleens could have twisted simultaneously. It is believed that among multiple spleens, those with looser fixation and longer feeding vessels are more prone to torsion. In all cases, the twisted spleen was surgically removed. In our patient, however, we also performed preventive fixation of another free spleen—an approach not described in the previous 10 cases.

**Table 1:** Eleven cases of splenic torsion with polysplenia syndrome, including the present case

Authors	Year	Sex	Age	Spleen position	Numbers of torsed/total spleens	Malformation of pancreas	Other malformations and disorders
Ackerman et al.^4)^	1982	Female	7 months	Right	1/3		AC, ML, IM, VSD
Griffiths and Marshall^5)^	1984	Female	23 years	Right	2/2		LL
Lachmann et al.^6)^	2006	Female	9 years	Right	1/ND		ML, RS
Rasool and Mirza^7)^	2011	Female	2 days	Right	1/7		LL, RS, jejunal atresia
Dash et al.^8)^	2013	Male	12 years	Right	1/5		LL, RS
Fujiwara et al.^9)^	2019	Female	10 years	Left	1/2		AVSD, BA, ML
Kubo et al.^10)^	2019	Female	21 years	Left	1/3		ND
Kennedy et al.^11)^	2021	Female	9 years	Left	5/7	AP	AC, IM, preduodenal PV
Draghmeh et al.^12)^	2022	Female	13 years	Left	1/5		AC, BBL, IM, ML, bicornuate uterus
Cheang et al.^13)^	2022	Male	8 years	Right	1/6		AC, ML, levocardia
Present case	2025	Female	30 years	Right	1/5	ADP	AC, BA, BBL, ML, RS, double IVC

AC, azygous continuation; ADP, agenesis of dorsal pancreas; AP, annular pancreas; AVSD, atrioventricular septal defect; BA, bradyarrhythmia; BBL, bilateral bilobed lung; IM, intestinal malrotation; IVC, inferior vena cava; LL, left-sided liver; ML, midline liver; ND, no description; PV, portal vein; RS, right-sided stomach; VSD, ventricular septal defect

As in the present case, there have been reports of polysplenia syndrome coexisting with agenesis of the dorsal pancreas.^18)^ However, among such cases, this is the 1st reported instance involving splenic torsion. Normally, the pancreas develops from 2 embryologic buds: a ventral anlage, which forms the uncinate process and pancreatic head, and a dorsal anlage, which gives rise to the body and the tail. Both the dorsal pancreas and the spleen develop within the dorsal mesogastrium, so concurrent anomalies in both organs can occur in patients with polysplenia syndrome.^18,19)^ Ongoing research into the relationship between agenesis of the dorsal pancreas and certain gene mutations may further clarify their association with polysplenia syndrome.^20,21)^

In cases of dorsal pancreatic agenesis, the fixation of vessels between the ventral pancreas and spleen may be compromised. However, in our case, although the ventral pancreas was in a normal position, the spleen was abnormally located on the right side. With the stomach also situated on the right, the stomach, duodenal bulb, descending portion of the duodenum, and pancreatic head were clustered in the upper right abdomen. Therefore, the splenic artery (SA) anatomy was likely abnormal as well, although it remains unclear whether the agenesis of the dorsal pancreas directly caused the torsion.

**Fig. 4:**
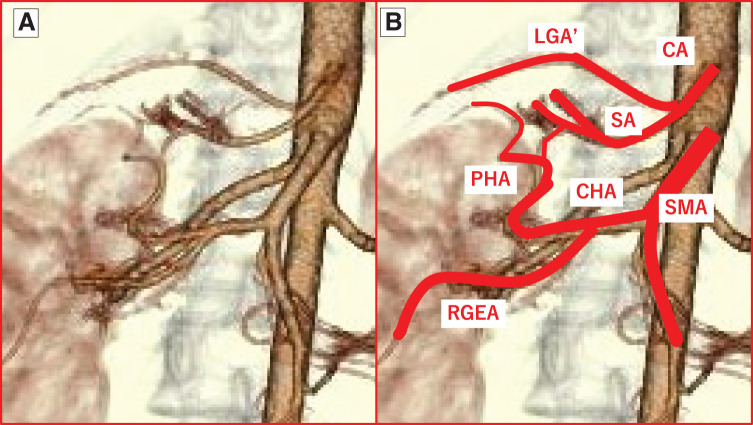
3D CT angiography of abdominal vasculature. (**A**) Front view after discharge. (**B**) The CA and SMA are highlighted with red lines. After coursing toward the right posterior side, the CA divides into an upper branch—presumed to be an LGA’—and a lower branch, the SA. The SMA divides into right and left branches. The right-hand branch represents the CHA, which further divides into upper and lower branches. The upper branch curves in an arc toward the head and continues as the PHA, while the lower branch passes caudally through the pancreatic head and becomes the RGEA. CA, celiac artery; CHA, common hepatic artery; LGA, left gastric artery; LGA’, variant left gastric artery; PHA, proper hepatic artery; RGEA, right gastroepiploic artery; SA, splenic artery; SMA, superior mesenteric artery

**Fig. 5:**
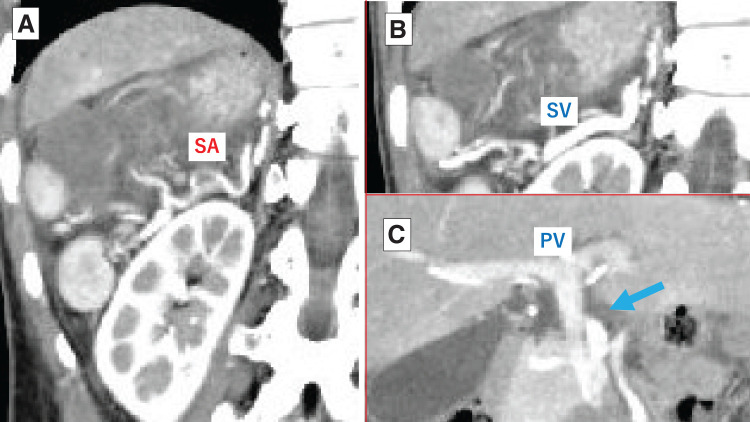
Contrast-enhanced CT on admission. (**A**) The SA branches into multiple vessels, forming an arterial arcade in the periphery that supplies each spleen. (**B**) The SV runs along the right dorsal side, anterior to the SA. (**C**) The SV joins from the left to form the PV (blue arrow). PV, portal vein; SA, splenic artery; SV, splenic vein

**Fig. 6:**
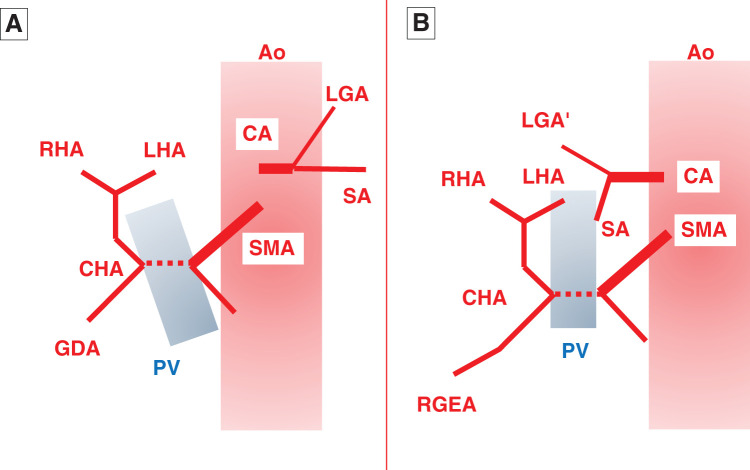
Schematic diagrams of the CA and SMA. (**A**) Type VI (group 24) of Adachi’s classification. The gastro-splenic and hepatomesenteric trunks independently originate from the Ao. The CHA passes dorsal to the PV and then divides into the PHA (which further branches into the RHA and LHA) and the GDA. (**B**) Current case. The gastro-splenic trunk, which courses to the right, and the hepatomesenteric trunk independently originate from the Ao. The CA runs rightward and then branches into an upper branch (LGA’) and a lower branch (SA). The CHA passes dorsal to the PV and divides into the PHA and the RGEA. Ao, aorta; CA, celiac artery; CHA, common hepatic artery; GDA, gastroduodenal artery; LGA’, variant left gastric artery; LHA, left hepatic artery; PHA, proper hepatic artery; PV, portal vein; RGEA, right gastroepiploic artery; RHA, right hepatic artery; SA, splenic artery; SMA, superior mesenteric artery

In fact, our patient exhibited 2 notable anatomical features. First, the complex variations of the celiac artery, superior mesenteric artery (SMA), and portal vein (PV) systems were clustered within the narrow space of the upper right abdomen. After coursing toward the right posterior side, the celiac trunk divided into an upper branch—presumed to be a variant left gastric artery—which supplied the proximal stomach, and a lower branch, the SA (**Fig. 4**). The SA appeared to branch into multiple vessels, forming an arterial arcade in the periphery and distributing blood to each spleen. The splenic vein ran along the right dorsal side, anterior to the SA, and joined from the left to form the PV (**Fig. 5**). The SMA divided into right and left branches. The right branch represented the common hepatic artery, which coursed along the dorsal side of the PV and then split into upper and lower branches on its right. The upper branch curved in an arc toward the head and became the proper hepatic artery, while the lower branch coursed caudally, passing through the pancreatic head before continuing as the right gastroepiploic artery. Except for the right-sided SA and the variant left gastric artery, this arterial pattern resembled Adachi type VI (group 24), reported in approximately 1.5% of normal individuals^22)^ (**Fig. 6**). Similar to the current case, 3 reports of heterotaxy syndrome have described the common hepatic artery arising from the SMA.^23–25)^ Matsuda *et al*.^25)^ suggested that this feature—specifically, the proper hepatic artery originating from the SMA—may be characteristic of patients with situs ambiguus accompanied by polysplenia syndrome. Notably, all of these cases, including ours, had spleens located on the right side, suggesting an association with a right-sided SA.

Second, among the 5 spleens, the centrally and dorsally positioned spleen (**Fig. 2**) was twisted and lacked fixation. Based on this, we hypothesize that splenic torsion in this case was caused by the floating central spleen and its relatively elongated SA. While each previously described patient may have had unique anatomical features, prior reports have not provided detailed descriptions of pancreatic or splenic vascular anomalies. Aside from our case, the only mention of coexisting pancreatic malformation was a case involving annular pancreas.^11)^ Going forward, it will be of great interest to analyze polysplenia syndrome with a focus on pancreatic anomalies and splenic vasculature. Such analyses may help identify patterns contributing to splenic torsion, potentially enabling preventive strategies in high-risk patients.

Acquired wandering spleen is more commonly observed in young women, and hormonal influences are considered as 1 contributing factor.^26)^ Thus, acquired factors cannot be ruled out in our case. When performing splenectomy, postoperative complications must be considered, especially overwhelming post-splenectomy infection. However, in this and other reported cases of splenectomy in polysplenia syndrome, many spleens were preserved, suggesting retained splenic function. Indeed, in patients with biliary atresia who underwent partial splenectomy, no fulminant infections were observed during long-term follow-up.^27)^ Nonetheless, because functional asplenia has been reported in a patient with polysplenia syndrome, careful long-term observation remains essential.^28)^

## CONCLUSIONS

We encountered a rare case of splenic torsion associated with polysplenia syndrome. To date, no prior literature has suggested a relationship between pancreatic malformations and splenic torsion in such cases. Accumulation of additional cases is needed to clarify the potential relationship between pancreatic malformations and splenic torsion, as well as to assess the long-term outcomes following splenectomy.
